# Evaluation of the efficacy of peer-learning method in nutrition students of Shiraz University of Medical Sciences

**Published:** 2014-04

**Authors:** MOHAMMAD REZA DEHGHANI, MITRA AMINI, JAVAD KOJURI, PARISA NABEIEI

**Affiliations:** Quality improvement in Clinical Education Research Center, Shiraz University of Medical Sciences, Shiraz, Iran

**Keywords:** Learning, Students, Teaching

## Abstract

**Introduction**: One of notable initiatives in improving the academic education is to use the abilities of students to learn together in a new and effective system of peer learning. In this regard, Education  Development Center in Shiraz University of Medical Sciences proceed to implementation and study of the curriculum and teaching methods course in the form of peer learning for college students of nutrition science and survey the efficacy of this implementation.

**Methods**: This study was conducted two parts: qualitative and quantitative survey. A quasi-experimental, pre test/post-test research was used in quantitative part. In this study, whole groups of undergraduate nutrition students in courses of study and learning techniques with the help of teachers held a course and took a part in a competition in 15 major subjects of study and learning methods. The study lasted for two-week sessions and whole of nutrition students were included. We used pair t test for comparison pre test and post test in this study.

**Results**: In the quantitative part of the study, the results showed a significant difference between pre-test (0.0346±0.108) and post-test scores (0.809±0.187) of the students. This means that the level of knowledge of students, who participated in this course, has significant difference before and after the peer learning course (pair t-test=1.010, p=0.002). The results of the quality survey of the training course also indicated satisfaction of participants and necessity of teacher’s presence and control at courses.

**Conclusions**: The results of this study confirm the results of the previous studies emphasizing numerous positive effects of the peer learning methods in the academic community. The results also suggest that peer learning is effective to enhancement of the students' confidence and learning. Peer learning also helps to develop their future responsibilities.

## Introduction

Peer learning method is a continuous part of human learning and is a teaching strategy in which non-professional teachers with different ages and same learning levels, help each other to learn from each other (1). One of the aims of peer learning is to increase the learning confidence the students in a supportive learning environment and to assist students to develop collaborative learning partnerships (2).

There are different types of peer learning. Peer learners may be at same or higher level in terms of education or experience; older students teach theoretical or practical lessons to younger students and usually is an effective and practical method (2-4).

The educational programs are that is conducted by peers have positive effects on the tutorial and people under the surveillance. In fact, acting as a peer tutor can be an appealing and constructive educational opportunity to further students' academic development (5). This method of learning is a useful way to prepare students for their future role as teachers of medical sciences. Several studies have proven the benefits of this method (6). Buckley and Zamora reported that peer education enables the student to overcome personal fears during the lecture (7).

One of the advantages of this method is increasing the confidence (8, 9) improved emotional-cognitive skills and conduction, (8) increasing the presentation skills, teamwork, decision making, responsibility, (10-11) developing the critical thinking skills, (12) and improved test scores. In several studies, is has been shown that using peer education improved academic functional outputs (13) It is also reported that this method of education can increase the cooperation between the groups to increase health information (14) and also, leads to better performance of the students (15-17).

The main aim of the peer teaching program at the university is development of practice, increase students' interest and participation in academic activities and also to improvement of the educational environment (18).

According to the effects of student peer teaching as a professor and also special attention of college to the design of student-centered curriculum in medical education, this study was conducted to evaluate the effectiveness of peer teaching and learning using college students of nutrition sciences in Shiraz University of Medical Sciences. 

## Methods

Thirty five under graduate nutrition students at Shiraz University of Medical Sciences were included in this study. The study was conducted under the supervision of peer tutoring program teachers. A quasi-experimental, pre test/post-test research was used in study. The study was done in two qualitative and quantitative parts. In the quantitative part, fifteen key issue in teaching and learning methods were evaluated including awareness about the principles and methods of learning and study notes, speed, time management, the role of concept mapping in learning, communication with friends, academic staffs and attendance, learning styles and motivation, scientific principles and practical strategies to improve learning memory, internet search methods, education technology, exam preparation, cooperative learning methods, cope with stressful conditions, six thinking hats and creativity in education and presentation in the class. This section was performed as 2 sessions per week for 35 first-year students in nutritional science.

Each group contained 2-3 students and they chose a certain subject based on their interests. This study has been conducted according to Associate for medical education in Europe (AMEE guild no 31) (10). Information transition pattern was from student to student and under teacher guide and in fact, teachers were only guiding of the group. Each group included two subgroups. Students at each subgroup evaluated various aspects of each mentioned topic and they taught training sessions to other members at other groups.

Each student acted as a teacher in one topic and as a student for other 14 topics. Before starting the course, a questionnaire containing 10 questions of the main subject (which was going to be taught during the course) as a pre-test was filled by all participants. All participants were asked to answer questions based on their previous knowledge.

At the end, the questionnaires were also filled by participants as a post-test. This comparing method of two tests was to exclude the confounding factor that caused the differences among participants knowledge.

The questionnaire included questions such as note taking strategies, concentrate memory, appropriate environment for study, understanding the lessons, answering to questions, effective communication barriers between teachers and students, professorial ways of coping with stress, learning strategies, creativity in education and professionalism. Content validity of the questionnaire was evaluated and confirmed by 5 members of the academic faculty of Education Development Center of Shiraz University of Medical Sciences and its reliability was confirmed by test/retest in one workshop held for learning and study strategies for a group of medical students using Cronbach's alpha by 83%. The survey questionnaire also included questions about the quality of the course as well as assessment of satisfaction of study. The questionnaire was scored based on a scoring style (from 1 to 5); 1 as very poor, 2 as poor, 3 as average, 4 as good and 5 as very good. The mean±SD scores of questionnaire were calculated and done by SPSS 14 (SPSS Inc, Chicago, IL, USA). In the second part of the study, semi-structured questionnaire was used including five questions concerning the nature quality of holding this learning method widely in all courses and for all students in different levels. In the second part of this study was semi-structured questionnaires was used among the students. At the end of the course, the questionnaire was filled out by all students and the organizers of the course, and all participants were asked to carefully comment about this course to improve the courses like this. SPSS software, 14, was used to analyze the collected data; the analysis of data was performed using paired t-test and descriptive statistics. P ≤0.05 was considered as significant difference.

## Results

In this study, 35 nutrition students including with exclusion of 3 non-compliant students, 17 female (53%) and 15 male (47%) participated in two, pre-test and post-test stages. The minimum age for students was 21 years old and maximum was 23 years. It is noteworthy to mention that until the end of the test phase, no samples were excluded. As explained in the methods section, the questionnaires were filled out in two stages: at beginning and end of the course. Descriptive analyses of the results are listed in Table 1.

**Table 1:** Descriptive statistics of pre-test and post-test results of evaluation of peer learning method in nutrition science under graduate students

**Questions**	**Mean±SD** **Pre-test**	**Mean±SD** **Post-test**
Note taking strategies	0.311±0.104	1±0.602
Concentrate memory	0.209±0.850	0.968±0.93
Appropriate environment for study	0.238±0.094	0.875±0.178
Understand the lessons	0.332±0.116	0.906±0.189
Answering to questions	0.200±0.079	0.906±0.189
Effective communication barriers	0.405±0.131	0.656±0.162
Professorial ways of coping to stress	0.420±0.134	0.718±0.172
Learning strategies	0.389±0.136	0.593±0.148
Creativity in Education	0.430±0.141	0.468±0.131
Professionalism	0532±0.152	1±0.202

We used pair t-test for comparison pre test and post test in this study and it was found that for nearly all scales, there is the statistically significant difference in the rate of students learning (pair t-test=1.010, p=0.002).

**Table2:** Comparison of mean scores of students before and after participating in the course

**Test**	**N**	**Min**	**Max**	**Mean±SD**	**Pair t-test**	**p**
**Pre-Test**	35	0.200	0.532	0.346±0.108	1.010	0.002
**Post-Test**	35	0.468	0.968	0.809±0.187		

Scores range is from 0 to 1

Also the results of the course learning survey from peer learning of the students participating in this study, showed an increase in confidence of participants, improvement in the stimulated learning, developments in future works and decrease in their anxiety and stress.

**Figure 1:**
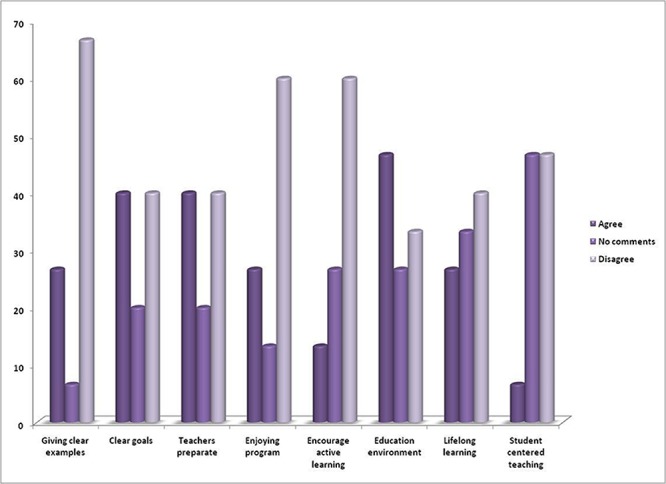
Students' comments about the quality of peer learning education course

The participants also believed that this course have had clear goals and strong management their comments in this regard are shown in Figure 1.

## Discussion

Peer-assisted learning (PAL) is an efficient way of incorporating extra training with which students can reinforce basic learning, which students find enjoyable and assess positively (19). The confidence of both trainers and trainees in examination technique was evaluated highly after these sessions. PAL allows trainers to assist their trainees with teaching and learning support and is implemented in many undergraduate and postgraduate programs (20, 21). Actually this method, cannot be a replacement for teacher absence or poor teaching in the college, but is an arena for interested students wishing to improve their abilities in required courses (16). Today, attention and emphasis on learning has changed to learning by teaching and many teachers focus on designing and maintaining a student-centered method through peer learning method instead of teaching a subject (22).

This study focused on student's teaching to others and to evaluate the effects of their teaching together. In one study in gastroenterology and hematology field, selected trainers learned more, gained better knowledge and had better retention of memory (10) and in another study, it was found that 86% of trainees wished to be educator (3). Of course, in this study, we provided this rotated opportunity to all of the students.

In this study, we evaluated the effects of peer learning methods as a tool to promote and enhance the students’ knowledge. The concept of peer learning has recently been considered among the students of the same age and peer education of teacher and student, as an organized training program in medical education. In this study, the aim is providing new graduates with teaching skills and experience (23) obtained for nutrition sciences.

As stated in methods section, this study was conducted in two parts. In the first part, there was a significant positive correlation between pre-test and post-test scores (p<0.05). It is likely that the 6 other subjects were more familiar for the students because they were similar to some of the topics at their high school, so they showed more learning skills and in 9 other issue due to lack of familiarity and because of a competitive aspect between learners and student groups that led to discussion and more attention, so better teaching contents were achieved.

Few studies have been conducted on this subject in our country. Mousavi surveyed comparative effectiveness of peer tutoring on academic achievement and anxiety and found that peer education had a significant effect on academic achievement and anxiety coping between two experimental and control groups (24).

Mehrabi also studied the effects of peer education on medical students and concluded that the students’ scores of the students in different clinical fields showed a significant increase compared with control group (p<0.05). Peer learning was effective in increasing the students' clinical skills and student roles as teachers led to a combination of attitudes, skills and knowledge in educators and learners (25).

Results of our study indicate the effectiveness of the training on the participants’ knowledge and attitudes, helping each other as partners in the learning process (26), evaluating the effect of peer learning student; (27) and studying peer learning strategies through peer to improve study skills in students (23).

But Walsh concluded that computer based education and training by skilled, is more effective than peer learning method. He found that external feedback and response is required as measurement tool, tutor specialization degree is a vital and effective factor, and determining the success of peer learning depends on the kind of evaluation method (28).

Wang also concluded that students who participated in learning from peers had higher scores in USMLE and GPA than other students. In Wang’ s study also an external criterion was considered as the measure pattern, but the result was in favor of peer learning methods. Unlike Walsh’s study (29), our study indicated that learning from peers is more effective on test scores, student satisfaction and personal and professional development.

Kimiai et al. concluded that despite higher scores in the intervention group, this difference was not statistically significant (30). In our study, results of the satisfaction survey indicated high levels of satisfaction among participants (approximately 88%). In other words, PAL in this course had been very pleasure and helpful.

In a study that investigated the characteristics of peer teaching on 447 final year medical students in Brazil, it was shown that this system provides engagement and lively educational opportunities for students' academic achievement and it can be very helpful to the choice of future profession (31). The participants in the present study also consider the proposed system as attractive educational opportunities.

In another study in Australia on 131 second-year medical students to evaluate the effectiveness of this method, it was shown that peer tutors can have an important role in helping each other and enhancing confidence and practical skills (32). These findings were similar with the results of our study.

In The West Australian Medical Students' Society (WAMSS), grand rounds are held and the students of which students of 4-6 years as a volunteer, teach clinical skills to other students. The participants of the course were extremely helpful and pleasant and the participants believed that this course could help them in clinical skills 3 times of other types of educations such as self-positive evaluation of abilities (33).

From the perspective of the students that participated in the study, teachers’ training and supervision has a significant effect in effective learning. They believe that in the absence of tutor and supervision peer education learning will not happen so well. For example, one student commented that: “The tutor in this course is very important so that if sometimes, if occasionally, errors occur by peer, teacher can realize quickly and remove it, or if a subject is not understood, the teacher can explain it again.

Students also believed that characteristics of special education in peers such as questioning the teacher morale, maintaining classmates respect during the education period and having good rhetoric in peers are the influential factors on learning through peer process. 

## Conclusions

The results of this study and previous studies are consistent with each other and all of them emphasize on numerous and constructive effects of such modern learning methods in the academic environment. The results of this study also suggest that peer-assisted learning increase confidence; reduce anxiety and stress and effect on development of learner’s future responsibilities. Also according to the students' comments in the qualitative part of the study, it seems that teachers' comments and detailed feedback to students during the course can increase the effectiveness of the course. Also, communication with other students during the course can increase the sense of cooperation and trust. Also, it is effective on enhancing and improving the environment of these courses. Educational authorities also can establish the necessary infrastructure to implement these courses with new methods of teaching, and therefore send efficient students and knowledgeable to the students’ national health system.

**Limitation**

This study is not a complete randomized controlled trial because it was not possible to divide students randomly to intervention and comparison groups. 
